# Oral health knowledge, attitudes, and practices among female public health and nutrition university students in Qatar

**DOI:** 10.3389/fpubh.2024.1405439

**Published:** 2024-10-31

**Authors:** Amal Elwadia, Aisha Naeem, Shajitha Thekke Veettil, Nikki Orquia, Diana Alsayed Hassan, Paul Amuna, Alaa Daud

**Affiliations:** ^1^Department of Clinical Research, Primary Health Care Corporation, Doha, Qatar; ^2^Research and Graduate Studies, QU Health, Qatar University, Doha, Qatar; ^3^College of Health Sciences, QU Health, Qatar University, Doha, Qatar; ^4^University of Health and Allied Sciences, Ho, Ghana; ^5^College of Dental Medicine, QU Health, Qatar University, Doha, Qatar

**Keywords:** knowledge, attitude, public health, nutrition, oral health, interprofessional education, undergraduate students, dental education

## Abstract

**Objectives:**

The present study aimed to determine oral health (OH) related knowledge, attitudes, and practices among Public Health (PH) and Nutrition (NU) students at Qatar University.

**Method:**

A cross-sectional study was conducted using a pre-validated questionnaire comprising 36 items covering demographics, knowledge, attitudes and perception of oral health practices. Data were analyzed descriptively (means, standard deviations, proportions) and inferentially using statistical tests including *t*-tests for comparing means, and chi-square tests for examining associations between categorical variables.

**Results:**

A total of 112 female undergraduate students participated, including 41 from PH and 71 from NU programs (response rate for both courses = 59.5%). The mean age was 21.8, while 23% were Qataris and 77% non-Qataris. Overall, students demonstrated good knowledge of OH (67.65%), with the PH group scoring higher (70.7%) than NU (65.35%). Knowledge regarding dental plaque was low for both groups (31.0%). Attitudes toward OH varied among participants. Most students reported practicing brushing with fluoridated toothpaste and demonstrated high knowledge regarding the association between poor OH and general health (95%).

**Conclusion:**

Overall, PH and NU undergraduate students exhibited relatively good knowledge of OH, however, demonstrated a range of attitudes and practices, including suboptimal ones. Integration of OH education into the PH and NU curriculum is warranted, along with enhanced interprofessional education to promote self-awareness and improve patient oral health outcomes.

## Introduction

More than a decade ago, the World Health Organization (WHO) Global Oral Health Program (GOHP) sought to promote awareness of oral health (OH) worldwide (1, 2), given its importance to general health and quality of life (3). Meanwhile, even in high-income countries, oral disease is still a major public health concern, and the burden of oral disease is growing in many low and middle-income countries (2). In 2011 in Qatar, a national survey based on WHO Oral Health Survey was developed and implemented (4). Secondary data was later analyzed of 1,124 6-year-old private and government primary school children, revealing nearly 7 out of 10 children aged 6-years-old suffered from dental caries lesions (5). In another study conducted in Qatar, a strong relationship was found between dietary habits and dental caries among female, overweight or obese subjects (6). It was concluded that promoting general health and OH awareness, and education on tooth brushing habits, adequate nutrition, and obesity prevention is pivotal to avoid dental caries among children aged 6–15 years old. Alkhtib et al. (7) also reported that dental caries among Qatari preschool children was related to a number of factors including frequent consumption of foods rich in carbohydrates.

Dental attendance patterns and barriers such as lack of awareness and knowledge, may be attributable to potentially modifiable factors such as behaviors and dental care (8, 9). Poor oral hygiene was found to be directly related to the level of knowledge, attitude and behavior of the person (10). A recent study investigating the impact of knowledge level of Qatari people toward oral health, deduced that people with higher levels of dental knowledge showed positive OH behavior, while people with lower knowledge levels showed less frequency of tooth brushing and avoided visits to dental clinics (11). Among young adults including students transitioning from their own home environment where they could be encouraged and monitored by their parents with relation to health matters, to a period of ‘independence’ in college, where they may live away from home, adhering to good health practices may be compromised, leading to OH problems (10). Several factors influence the OH practices of university students, including knowledge, attitude, and behavior toward OH. For example, a study in Saudi Arabia revealed that despite high oral hygiene habits, poor dental attendance and health-risk behaviors such as smoking, and lack of exercise affected OH outcomes (12). Additionally, studies have shown that OH knowledge significantly impacts oral hygiene behaviors, with dental students demonstrating superior practices compared to non-dental students (13). Socioeconomic factors and stress levels also play a role, as higher stress can lead to increased snacking and poor dietary habits, further affecting OH (14). In Qatar, there is growing interest in the overall health of the population, including OH. Therefore, it is crucial to explore the knowledge, attitudes, and practices of such young college cohorts toward OH, to prevent oral disease.

Health care professionals, including those working in the fields of public health and nutrition, can play an important role in the OH education of their patients, families, and the wider community. The role of a nutritionist is to council individuals on nutritional issues and healthy eating habits, develop meal and nutrition plans and evaluate the effect of nutrition plans on patients (15, 16). The relationship between nutrition and OH is deeply interconnected. Nutritional deficiencies can lead to OH issues, such as increased susceptibility to periodontal disease, caries, and other oral infections, which can subsequently impact dietary intake and nutritional status. For instance, tooth loss or oral pain may impair one’s ability to chew food properly, leading to nutritional deficiencies. A balanced diet rich in essential nutrients is necessary to maintain the structural and functional integrity of oral tissues, including the production of saliva and overall oral immunity (17, 18). Conversely, OH care following diet consumption is crucial due to the direct impact of dietary habits on dental conditions such as caries, erosion, and periodontal diseases. The consumption of foods high in sugars, acids, and fermentable carbohydrates promotes the growth of harmful bacteria in the mouth, contributing to tooth decay and enamel erosion (19). Proper OH practices, such as brushing, using dental floss, and rinsing the mouth after meals, help mitigate these effects. Additionally, the inclusion of cariostatic foods, like cheese or xylitol-containing gum, can enhance OH after meals by stimulating saliva production and neutralizing acids (20). It is therefore essential for future nutrition professionals to have a basic understanding of OH because their patients’ nutritional status directly impacts oral health and vice versa. By understanding this relationship, nutrition professionals can guide patients in choosing diets that promote both systemic and oral health and collaborate with dental professionals to provide comprehensive care (21). On the other hand, public health professionals analyze and develop programs that protect the health of individuals, families and communities (22). It is therefore important to recognize the level of their own knowledge, their attitudes, and behaviors toward oral health. The attitudes and behaviors of healthcare professionals toward oral health maintenance are strongly influenced by their understanding of preventive OH measures, which is crucial for enhancing their patients’ OH in the future. Studies have shown that healthcare providers with positive attitudes toward preventive dentistry tend to engage in better OH practices, which not only improves their own OH but also enhances their ability to educate and motivate patients to adopt similar preventive measures (23). Furthermore, healthcare professionals who exhibit better oral self-care habits are more likely to influence their patients to adhere to preventive OH measures, thereby improving overall patient outcomes (24). This connection underscores the importance of incorporating OH education into the curriculum for healthcare professionals, as their personal OH habits directly impact their patients’ health behaviors (25). Health science college students may have the opportunity to engage in OH educational activities including raising awareness, focusing on the frequency of tooth brushing for example, and the consumption of low-sugar healthy diets. These could be promoted by carrying out campaigns in schools, mass media, and advocacy for legislations for food composition, etc. to help prevent and reduce the incidence of dental caries in the community. It is for these reasons that public health and nutrition students were of particular interest for this study.

Given the nature, extent, and seriousness of OH problems among young people, this study was designed to help understand the situation with respect to the knowledge, attitude and practice of Public Health (PH) and Nutrition (NU) college students at Qatar University. Currently, no courses are taught within these programs covering any OH related topics.

Up to our knowledge, this is the first national study targeting health care professional students in the State of Qatar. Therefore, this will help provide baseline data and identify any gaps, in order to inform future studies and recommendations for improving the knowledge and understanding of OH issues and ensuring better practices for improved OH outcomes.

## Research methodology

### Study design

This descriptive cross-sectional study applied a survey on students of the two health-related programs, PH and NU. For the propose of this research study, approval from the Institutional Review Board (IRB) at Qatar University was obtained prior to commencement of the project (QU-IRB 1026-EA/19).

The study was conducted at Qatar University (QU), College of Health Sciences. QU is a leading public university in Qatar with a total student population over 23,000 students. The College of Health Science offers both Public Health and Nutrition programs. In this study, we exclusively included female participants due to the specific enrollment policies of the College of Health Sciences at Qatar University, which only admits female students into the Public Health and Nutrition programs. The sampling frame was drawn from all female undergraduate students pursuing the four-year Bachelor of Science degree in Public Health (PH) and the Nutrition (NU) programs. At the time of the study, the total enrolled female student population across both programs, spanning from year one to year four, was 188 (98 in NU and 90 in PH).

According to Cochran’s formula for calculating the sample size for proportions, for a population of 200 at a precision of 7 and 5% alpha at 95% confidence interval, the minimum number needed for statistical inference was 101 participants. This calculation factored in an assumed dropout rate of 10%. Ultimately, the study enrolled 112 participants to ensure adequate statistical power for analysis. The sampling process and distribution of the survey instrument were managed by an Administrator within the College.

All female students enrolled in years 1–4 of either PH or NU undergraduate program were eligible to participate in the study. Students were approached in class and briefed about the study with participant information sheets disseminated. Its purpose was to explain the aim of the study, help potential participants to decide whether they want to take part and to provide relevant details, including a summary of any risks and benefits to the participant, what will happen to the participant’s data, and contact details. This was conducted by a trained research assistant capable of answering any queries. It was emphasized that participation in the study was completely voluntary and prior consent was obtained from all the participants. Following informed consent, hard-copy surveys were distributed to all participants by an impartial administrator not affiliated with the research to mitigate any potential biases during the data collection process. Reassurance was given to all participants that their responses would remain anonymous, with no identifiable information collected, and that confidentiality would be maintained throughout the study. Any personally identifiable information (PII) that could directly or indirectly identify participants was removed and replaced with participant codes, stored in encrypted computer-based files and only shared with authorized personnel.

### Data collection

A structured self-administered questionnaire (SAQ) on knowledge, attitude and practices toward OH with 36 questions was used. The questionnaire was adopted and modified by the authors from originally validated questionnaires (26–28) and reviewed by educational experts in the field. The modified questionnaire consisted of four parts. The first part was about demographic questions-gender, age, nationality, and level of study. The second part had questions assessing information, with response options being accurate or inaccurate knowledge or do not know. The attitude questions in the third part, featured diverse descriptive options, namely, the reasons for visiting the dentist, including options such as seeking advice, addressing clinical complaints, undergoing treatment, scheduling routine check-ups, or indicating uncertainty.

The fourth part comprised two sets of practice questions. One set utilized a 5-point Likert-scale, offering options ranging from ‘excellent’ to ‘do not know’, while the other set investigated the consumption of various products, providing frequency percentages (%) per day, per week, and per month.

### Data analysis

All data items were coded and compiled into an Excel© database for systematic organization. Quantitative data were inputted into Excel© (Microsoft 365 version 2404) and subjected to comprehensive analysis. Descriptive statistics were employed to address the research inquiries, while inferential statistics were utilized to assess discrepancies between the two student groups, such as comparing mean ages.

Furthermore, all statistical analyses were conducted using R Studio (R version 4.2.3). Statistical tests such as Chi square were employed to investigate potential significant variations in consumption frequency across different food items. Fisher’s Exact Test was utilized when the sample size was small or expected frequencies were low. The significance level was set at *p* < 0.05. The SPSS software (v.23, IBM, Chicago, IL, USA) package was used for data analyses.

## Results

### Demographics

A total of 188 students were approached (90 PH and 98 NU), whereby 41 PH and 71 NU agreed to participate (total response rate = 59.6%), with a partial response rate among PH students being 45.6 and 72.4% among NU as shown in Table 1.

**Table 1:** Demographic profile of participants.

No of participants	Public health *N* = 41	Nutrition *N* = 71	*p* value
Average age	22.12 SD 2.15	21.63 SD 1.67	0.95
Nationality			
Qatari	12	14	0.25
Non-Qatari	29	57	
Grade			
Year 1	4	6	0.73
Year 2	5	15	
Year 3	8	23	
Year 4	24	27	

SD = Standard deviation.

The majority of students (76.7%) were of non-Qatari nationality. The average age of participants was 21.8 (±1.86) with no significant difference between the NU (21.63, ±1.67) and PH students (22.12, ±2.15) (Table 1).

The chi-square test was conducted to examine the association between nationality (Qatari vs. Non-Qatari) and the program enrolled (PH vs. NU) among the participants (Table 1). The data does not provide convincing support that nationality (Qatari vs. Non-Qatari) is associated with the choice of program (PH vs. NU) among the participants in this study (*χ*^2^ = 2.22, *α* = 0.05).

Table 1 also shows the demographic profile of the participants and the distribution according to year group. The largest group was the fourth-year students (45%), followed by third year (28%), second year (18%), and first year (9%). We observed a significant relationship (*χ*^2^ = 8.817, α =0.05), between the program enrolled and academic level among the participants (Table 1).

### Oral health knowledge

The findings in the present study suggest a relatively good overall level of OH knowledge (67.93%), and although PH students seemed to have better OH knowledge (70.73%) compared to the NU students (65.36%), the difference was not statistically significant (*p* = 0.587). Only 41% of participants (Table 2) knew the total number of teeth in a human dentition (42.3% of PH and 41.1% of NU students), however, in relation to knowing the exact difference in numbers between deciduous and permanent teeth, more PH students (58.5%) than NU students (46.5%) provided an accurate answer. Among all participants, there was little knowledge regarding the meaning of dental plaque among all participants (30%) with PH students faring a little better (36.6%) than the NU ones (25.4%). The majority of participants (88.4%) displayed good knowledge regarding the purpose of tooth brushing, with more NU students (88.7%) than PH students (80.5%) knowing about the importance of brushing teeth. Similarly, a high proportion of participants (74%) had good knowledge of the meaning of bleeding gums, with PH students (80.5%) showing better knowledge in this regard than NU students (70.4%) (Table 2). There was a moderate overall level of knowledge of the effects of fluoride on teeth (67.4%), reasons for developing oral cancer (71.25%), and the possibility of correcting irregular teeth (68.85%). All groups knew about the effects of sweets on teeth (92.2%) with all public health students (100%) answering correctly compared to 84.5% of nutrition students. Most respondents knew about the effects of poor OH on their general health (94.6%) with all PH students answering correctly, compared to 91.5% of NU students. The majority of both groups also knew about the effects of fluoride on teeth (65%) and predisposing factors for oral cancer, such as tobacco chewing and smoking (71%). Participants also demonstrated familiarity with correcting irregular teeth such as using braces (69.6%), with NU students (71.8%) showing slightly greater awareness compared to PH students (65.9%) as shown in Table 2.

**Table 2:** Distribution of participants’ knowledge of oral health issues.

Questions	Participant response	Public health *N* (%)	Nutrition *N* (%)	Total *N* (%)
Number of human dentitions.	Accurate answer	16 (39.0)	30 (42.2)	46 (41.1)
	Inaccurate answer	3 (7.3)	9 (12.7)	12 (10.7)
	Do not know	22 (53.7)	32 (45.1)	54 (48.2)
Total number of deciduous and permanent teeth.	Accurate answer	24 (58.5)	33 (46.4)	57 (50.9)
	Inaccurate answer	7 (17.1)	19 (26.8)	26 (23.2)
	Do not know	10 (12.4)	19 (26.8)	29 (25.9)
Purpose of tooth brushing.	Accurate answer	33 (80.4)	63 (88.7)	96 (85.7)
	Inaccurate answer	6 (14.6)	4 (5.6)	10 (8.9)
	Do not know	1 (2.4)	2 (2.8)	3 (2.7)
Meaning of dental plaque.	Accurate answer	15 (36.6)	18 (25.3)	33 (29.4)
	Inaccurate answer	25 (61.0)	30 (42.3)	55 (49.1)
	Do not know	1 (2.4)	23 (32.4)	24 (21.4)
Meaning of gum bleeding	Accurate answer	33 (80.4)	50 (70.4)	83 (74)
	Inaccurate answer	3 (7.3)	11 (15.4)	14 (12.5)
	Do not know	4 (9.7)	10 (14.0)	14 (12.5)
Effect of sweet on teeth.	Accurate answer	41 (100.0)	60 (84.5)	101 (92.2)
	Inaccurate answer	0 (0.0)	3 (4.2)	3 (2.7)
	Do not know	0 (0.0)	8 (11.3)	8 (7.1)
Effects of fluoride on teeth.	Accurate answer	31 (75.6)	42 (59.1)	73 (65.2)
	Inaccurate answer	7 (17.1)	22 (31.0)	29 (25.9)
	Do not know	3 (7.3)	7 (9.9)	10 (8.9)
Effect of oral health on general health.	Accurate answer	41 (100.0)	65 (91.5)	106 (94.6)
	Inaccurate answer	0 (0.0)	1 (1.4)	1 (0.9)
	Do not know	0 (0.0)	5 (7.1)	5 (4.5)
Reasons for oral cancer.	Accurate answer	29 (70.7)	51 (71.8)	80 (71.4)
	Inaccurate answer	3 (7.3)	2 (2.8)	5 (4.5)
	Do not Know	9 (22.0)	18 (25.4)	27 (24.1)
Possibility of correcting irregular teeth.	Accurate answer	27 (65.9)	51 (71.8)	78 (69.6)
	Inaccurate answer	3 (7.3)	2 (2.8)	5 (4.5)
	Do not know	11 (26.8)	18 (25.4)	29 (25.9)
Overall knowledge.	Accurate answer	290 (71.07)	464 (65.6)	754 (67.50)
	Inaccurate answer	57 (14.23)	103 (14.67)	160 (14.32)
	Do not know	61 (14.95)	142 (20.03)	203 (18.17)

The chi-square test of independence was performed to examine the relation between overall OH knowledge and group of students. The relation between these variables was not significant (*χ*^2^ = 4.86, *p* = 0.087). A significant association between the groups (public health students and nutrition students) and the type of answers was observed only for two variables, i.e., the “Meaning of dental plaque” (*p* = 0.009) and the “Effect of sweets on teeth” (*p* = 0.02). This suggests that public health students and nutrition students differed significantly in their understanding of these topics. For the remaining questions, the responses were similar between the groups, indicating no significant differences in their knowledge or perceptions on those aspects.

### Oral health attitudes

In total, only eight participants (7.1%) described the state of their teeth as excellent. Forty-one participants (36.6%) described their dental condition as good or very good, whereas 31.3 and 17.9% rated the state of their teeth as either average or poor, respectively (Table 3). Additionally, 7.1% of respondents were unable to assess the state of their teeth. Participants were also requested to describe the condition of their gums. A notable portion of both PH and NU rated their gum condition as either good or very good (29.5%) to excellent (18.8%), while 28.6% described them as average, 14.3% thought they were poor or very poor, and 8.9% in total were unsure about their gum health. Concerning the presence of any gum disease, the majority (65%) of our participants did not believe or know they had any gum disease, while 15% of respondents stated they had gum disease. However, almost 20% of all respondents did not know whether they had gum disease or not (Table 3). There were no statistically significant differences among respondents.

**Table 3:** Participants’ perception about oral health issues (*n* = 112).

Oral health status	Type of response	Public Health *N* (%)	Nutrition *N* (%)	Total *N* (%)
Participants’ description of the state of their teeth	Excellent	4 (3.5)	4 (3.5)	8 (7.1)
	Good/very good average	15 (13.3)	26 (23.2)	41 (36.6)
	Average	20 (17.8)	15 (13.3)	35 (31.3)
	Poor/very poor	14 (12.5)	6 (5.3)	20 (17.9)
	Do not know	3 (2.6)	5 (4.4)	8 (7.1)
Participants’ description of the state of their gums	Excellent	7 (6.2)	14 (12.5)	21 (18.7)
	Good/very good average	15 (13.3)	18 (16)	33 (29.5)
	Average	13 (11.6)	19 (16.9)	32 (28.6)
	Poor/very poor	6 (5.3)	10 (8.9)	16 (14.3)
	Do not know	4 (3.5)	6 (5.3)	10 (8.9)
Participants’ perception if they have gum disease	Yes	10 (8.9)	7 (6.2)	17 (15.2)
	No	41 (36)	32 (28.5)	73 (65.2)
	Declined to answer	0 (0.0)	0 (0.0)	0 (0.0)
	Do not know	13 (11.6)	9 (8)	22 (19.6)

While 51.21% of PH students reported having seen a dentist in the last 6 months, only 38.03% of nutrition students had seen a dentist over the same time period. Similarly, more public health students (26.83%) than nutrition students (19.72%) attended dental appointments over the previous 12 months and of the latter group, a further 19.73% had a dental appointment between the last 12–24 months (Table 4).

**Table 4:** Participants attitudes and practices toward oral health issues (*n* = 112).

Attitudes and experience	Type of response	No. of participants - public health *N* (%)	No. of participants - Nutrition *N* (%)	*p* value
Use of toothpaste	With fluoride	28 (68.3)	44 (62.0)	0.797
	Without fluoride	2 (4.9)	4 (5.6)	
	No	11 (26.8)	23 (32.4)	
	Do not know	0 (0)	0 (0)	
Participant’s last dental visit (Months)	<6	21 (51.2)	27 (38.0)	0.326
	6–12	11 (26.8)	14 (19.7)	
	>12 but <24	3 (7.3)	14 (19.7)	
	2 yrs to <5 yrs	4 (9.8)	10 (14.1)	
	≥5 yrs	2 (4.9)	4 (5.7)	
	Never	0 (0)	2 (2.8)	
Reason for dental visit	Consultation/advice	4 (9.8)	6 (8.5)	0.88
	Clinical complaint	14 (34.1)	31 (43.7)	
	Treatment/follow up	13 (31.7)	18 (25.3)	
	Routine check-up	9 (22)	15 (21.1)	
	Do not know	1 (2.4)	1 (1.4)	
Receipt of blood sugar advice from a dental health professional	Yes	3 (7.3)	2 (2.8)	0.2
	No	36 (87.8)	64 (90.1)	
	Declined to answer	0 (0)	1 (1.4)	
	Do not know	2 (4.9)	4 (5.7)	

Reasons for dental visits included acute complaints (34.15% PH vs. 43.66% NU students) or treatment and follow-up in 31.71 and 25.35% of PH and NU students, respectively. A similar number in both groups (21%) saw the dentist for a routine check-up. More PH students (51.21%) than NU students (38.03%) had visited a dentist in the last 6 months. A further 26.83% of PH compared to 19.72% of NU students had visited a dentist between 6 and 12 months. Therefore, over the last 12 months, 78% of PH students saw a dentist while only 57.7% of NU students saw a dentist within the last 12 months (Table 4). Regarding tooth brushing practices and the use of toothpaste (whether with or without fluoride), 68.2% of PH and 62% of NU students admitted using fluoride toothpaste while around 5% of both cohorts used non-fluoride toothpaste and around a third reported not using toothpaste when brushing their teeth.”

### Oral health practices

**Figure 1:**
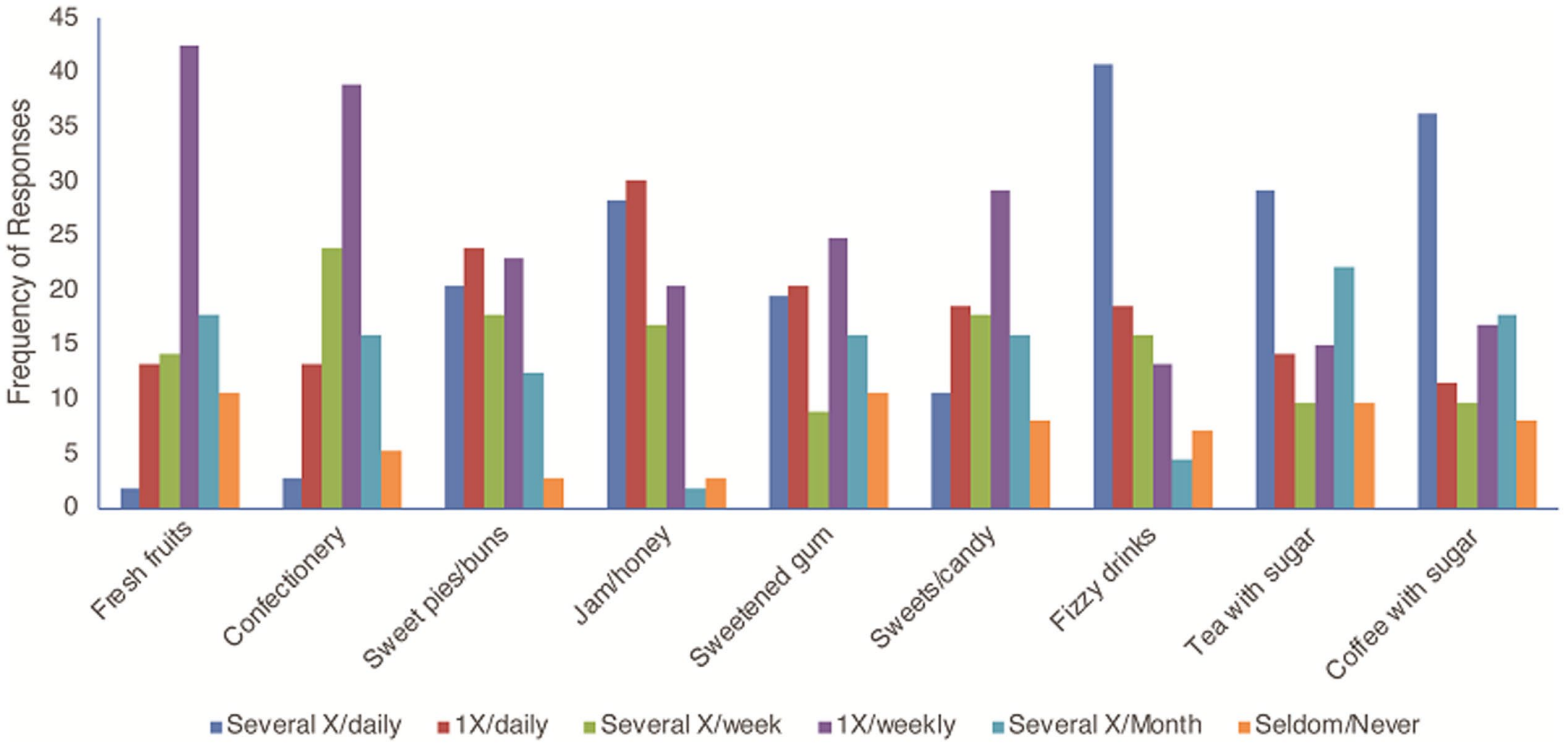
Relationship between participants’ oral health practices and the percentage of consumption of different foods, drinks and snacks. The chi-square test examined the association between consumption frequency and preferences for nine food items. The significant chi-square statistic (*χ^2^* = 774.79, *p* < 0.001) indicates a strong relationship between how often individuals consume certain foods and their preferences.

With regards to diet, very few participants (1.8%) consumed fresh fruit several times a day, compared to 10% who seldom or never took any fresh fruits. The majority of those who took fresh fruits had them only once weekly (42.5%). Others took them once a day (13.3%) or several times a month (17.7%) (Figure 1).

Concerning the frequency of sugar consumption, some participants consumed sugary products such as fizzy drinks (40.7%), coffee with sugar (36.3%), tea with sugar (29.2%), sweet pies and buns (20.4%), jam and honey (28.3%), sweetened gum (19.5%) and sweets or candy (10.6%) several times per day. Daily consumption of sugar-sweetened products was 59.3, 58.4, 47.8 44.3, 43.4, 39.9, 29.2 and 16%, respectively, for fizzy drinks, jam/honey, sugar-sweetened coffee, sweet pies and buns, sugar-sweetened tea, sweetened gum, sweets and candy and confectionery. Only 2.7% of participants seldom or never consumed sweet pies and buns, and sweetened gums; and 5.3% seldom took confectionery (Figure 1).

**Figure 2:**
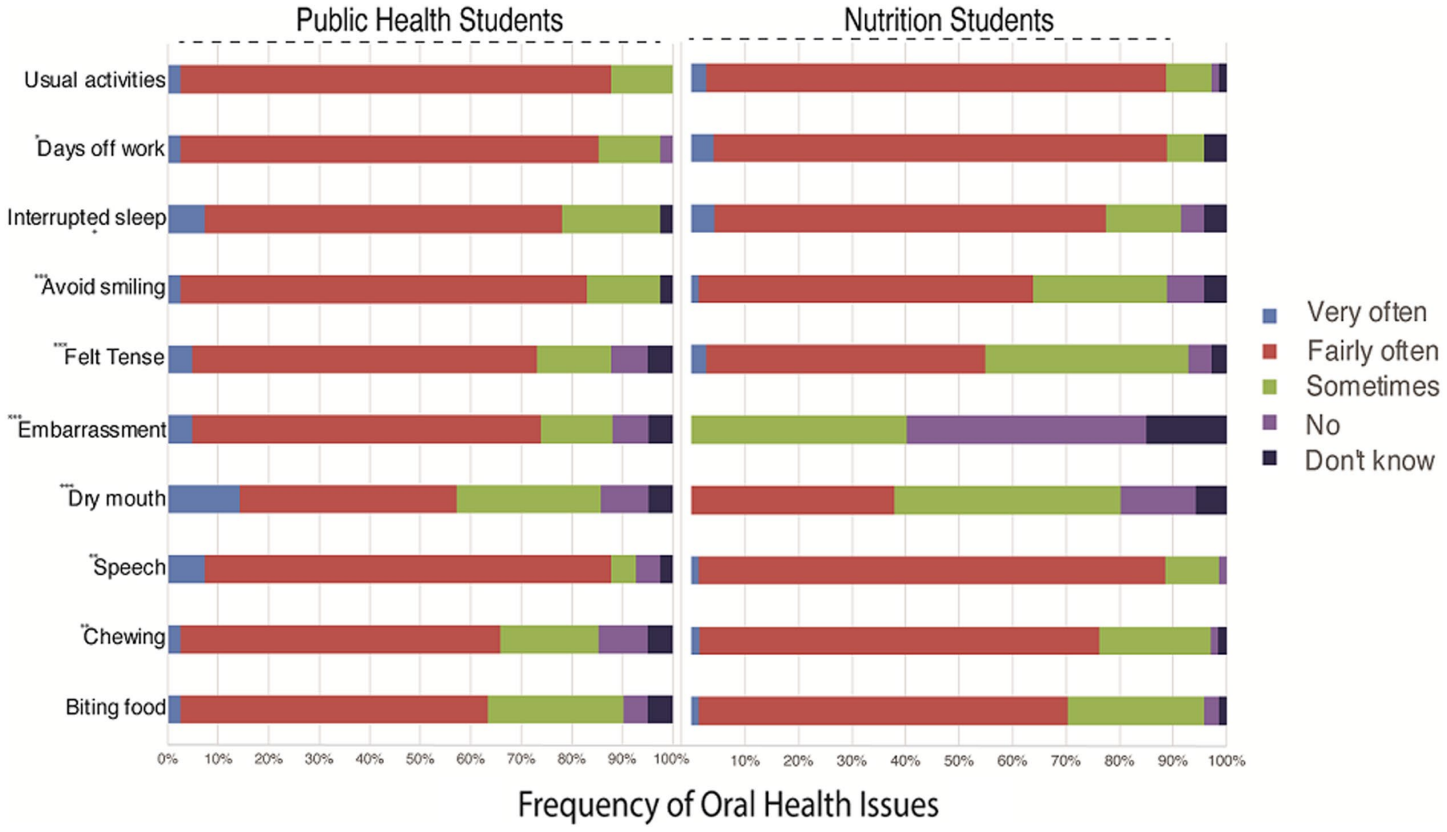
Challenges faced by participants regarding oral health issues over the past 12 months.

Data comparing the perspectives of PH and NU students on the impact of OH issues provide detailed insights into the prevalence and effects of these problems within their respective disciplines (Figure 2). Public Health students more frequently reported experiencing OH challenges, with approximately 60.96 to 85.37% indicating difficulties such as impaired speech, feelings of stress, and disruptions to daily activities. In contrast, while Nursing students acknowledge similar concerns, they generally report lower frequencies, ranging from approximately 38.03 to 85.92%. Nevertheless, there were no statistically significant differences among respondents. These findings underscore the interdisciplinary nature of oral health, suggesting potential areas for targeted interventions and collaborative efforts between PH and NU disciplines.

Notable findings emerge from our study, notably in chewing and dry mouth, where NU students report more chewing issues (*p* < 0.01) while PH students experience more dry mouth (*p* < 0.01) (Figure 2). Furthermore, NU students exhibit lower levels of embarrassment (*p* < 0.01) and tension (*p* < 0.01) regarding OH compared to their PU counterparts. Conversely, Nutrition Students report avoiding smiling more frequently (*p* < 0.01), indicating potential differences in self-image perceptions. While not statistically significant, trends suggest PH students may take more days off work (*p* = 0.08) and experience greater disruption to usual activities (*p* = 0.4714) due to OH concerns (Figure 2).

In general, difficulties experienced across both groups due to OH problems included biting on food (83.93%), chewing (50%), speech (59.82%), dry mouth (58.03%), embarrassment (69.64%) and tension (58.03%). Other difficulties include avoiding smiling (69.64%), interrupted sleep due to dental problems (72.32%), taking days off work (84.82%) and being unable to carry out usual activities (85.71%). Comparisons between the two student groups showed differences in their experiences relating to biting (60.96% vs. 69.1%), chewing (63.41% vs. 70.57%), speech (80.48% vs. 85.92%), dry mouth (21.95% vs. 38.03%), tension (68.29% vs. 52.12%); avoiding smiling (80.49% vs. 63.38%), interrupted sleep (70.73% vs. 73.24%), taking days off work (82.93% vs. 85.92%) and unable to carry out usual activities (85.37% vs. 85.2%) for PH and NU students, respectively. The comparisons showed that more nutrition students complained of difficulties than PH students except in the case of feeling embarrassed, where no nutrition student reported such difficulty, whereas 70.73% of PH students reported embarrassment. However, there were no statistically significant differences among PH and NU respondents.

## Discussion

This study sought to investigate knowledge, attitude, and OH practices among female PH and NU college students. This was a cross-sectional study involving self-reported responses from the participants.

The average age of female respondents was 21 years old, similar to the age ranges quoted in other studies, despite referring to male and female genders (29–31) reflecting the typical age range for most undergraduates. The distribution of nationalities between Qatari (23.2%) and non-Qatari (76.7%) is also reflective of the wider population of Qatar in which the indigenous Qatari population makes up about only 13% of the total population (11). In other studies, a common finding was that female students tended to have better OH knowledge and/or were more conversant with OH issues, and also more likely to utilize the services of a dental health practitioner, which could be attributed to the tendency of female students to place greater emphasis on the esthetic appearance of their teeth compared to their male counterparts (27, 32, 33). A recent study found notable discrepancy between self-perception of OH and clinically assessed oral health,” among female university students (34).

A closer look at the responses shows that the level of knowledge varied, depending on the subject area. For instance, only 41.1% of participants had good knowledge regarding the number of teeth in a human dentition and just under 51% knew the exact number of deciduous teeth. A study conducted in Germany in a population aged between 22 and 80 years old found that majority of the respondents (63%) recognized that poor OH can lead to gum disease, while 59.1% were aware that maintaining oral hygiene using toothpaste and a toothbrush is beneficial. Additionally, a significant proportion (81.5%) acknowledged the importance of regular dental visits, and 94.9% believed that regular tooth brushing helps prevent dental decay (16).

Interestingly, a similar study evaluating OH knowledge, attitude and behavior of male & female dental students found the correct answer for the meaning of gum bleeding, effect of fluorides on teeth and reasons for oral cancer to be 72, 81 and 78%, respectively (14). In that respect, findings indicate that the knowledge of PH and NU students in the current study was not far off compared to dental students, despite dental students receiving regular education regarding OH in their undergraduate curriculum. This could be due to the effects of media hype on OH care (20). In public health, the Centers for Disease Control and Prevention is actively using social media (21), and it has been identified that social media has a high impact in changing OH behaviors (22). This could explain the good number of accurate responses in this study in the aforementioned fields.

Participants demonstrated a high level of knowledge on topics related to the purpose of tooth brushing (84.7%) although one would have expected even better scores from healthcare students. The majority accurately knew about the effects of sweets on teeth (92.5%) with all the PH students responding correctly, while 84.5% of NU students did. One would expect a better score for the NU students given the fact they study diet and the effect of sugar on the body, however, they may not be studying the direct effect of sweets and sugary diet on teeth, presenting a gap in knowledge. Results from another study suggest that dietitian male & female college students lack sufficient knowledge on the relationship of diet and nutrition to oral health, indicating that collaborative efforts are essential between dental and dietetics professionals for educating consumers throughout the lifecycle (23).

The American Dental Association pointed out that a bidirectional relationship exists between OH and diet and nutrition. The latter affects the health of the tissues in the mouth; and the health of the mouth affects nutrients consumed (24). Therefore, nutrition college students must be educated on oral health, empowering them to relay this message to patients along with any nutritional advice.

In terms of the effect of OH on general health, all PH students had accurate knowledge and 91.5% of NU students did. Many systemic diseases are related to oral conditions and, thus, general health requires efforts of various healthcare professionals to prevent and control them (14, 25).

On analyzing the students’ knowledge about dental plaque, the findings of this study seem to be similar to reports from other Gulf countries and similar studies conducted among college students (15, 26, 27). In one study conducted in Saudi Arabia, the students had better knowledge about dental plaque but the main difference to this study is that the study population consisted of dental, medical, nursing and pharmacy students (32) and therefore, one would expect that they would have better knowledge in this field compared to PH and nutrition students in the current study.

Regarding attitudes toward tooth brushing and toothpaste (whether with or without fluoride), 68.2% of PH and 62% of NU students admitted using fluoride toothpaste while around 5% of both cohorts used non-fluoride toothpaste and around a third reported not using toothpaste when brushing their teeth. These results are expected given the fact that 75.6% of PH students and only 59.2% of NU students appeared to know the effect of fluoride on teeth in this study. Another reason for avoiding fluoride could be the claims of a relationship between fluoridation and adverse health outcomes, although findings provide no evidence that higher levels of fluoride (whether natural or artificial) in drinking water lead to greater risk of either bone cancer (osteosarcoma) or Ewing sarcoma (35–37). Other studies however have suggested that exposure to fluoride could be biologically associated with osteosarcoma since fluoride is deposited in bone and known to increase cell division (38, 39). To date, there is no strong evidence that higher levels of fluoride (whether natural or artificial) in drinking water or toothpaste may lead to a greater risk of osteosarcoma or other health-related conditions (40–42).

Regular dental visits play a crucial role in preventing, controlling and managing oral diseases (43–45). They contribute significantly to raising awareness and educating patients about oral health. A recent study underscored that daily tooth brushing, being toothache-free, and avoiding soft drinks were significantly associated with routine dental visits. Based on the findings, the authors suggests implementing initiatives to encourage routine dental visits, emphasizing the importance of preventive measures to reduce OH issues (45). In this study, it was observed that PH students exhibited higher rates of dental visits compared to NU students, indicating a potential gap in awareness.

Interestingly, a considerable percentage of students visited the dentist primarily due to dental complaints, reflecting a reactive rather than proactive approach to oral health. These findings are comparable to another study (33.9% participants), though from male students at various colleges (16). Neglecting OH can lead to productivity hindrances and affect quality of life.

The second most common reason for visiting the dentist was to receive dental treatment, followed by a routine check-up. Surprisingly, over 80% of students from both groups reported frequently taking time off or avoiding activities due to mouth-related issues. It is previously documented that neglecting OH can lead to suffering and pain, which can hinder productivity and affect the individuals’ quality of life (46). These findings underscore the importance of raising awareness and encouraging health profession students to maintain good oral hygiene habits and visit the dentist regularly. Encouraging regular dental visits not only benefits students’ own OH but also equips them to educate their future patients and prevent oral diseases.

Our study examined the relationship between consumption frequency and food item preferences using a chi-square test. Data from respondents regarding their consumption habits of nine food items were analyzed, revealing a significant association between consumption frequency and food preferences (*χ*^2^ = 774.79, *p* < 0.001). This suggests that individuals’ preferences for certain food items are influenced by how frequently they consume them. For instance, frequent consumers of fizzy drinks and sweets/candy tended to report consuming them several times daily, while those who consumed Fresh fruit did so several times a week or less frequently. These findings underscore the importance of considering consumption frequency when studying dietary habits and suggest avenues for further research into the underlying factors driving these associations and their implications for public health.

Implementing oral health-related education programs embedded within the PH and NU undergraduate curriculum can be reinforced within clinical practice. Additionally, integrating interprofessional education (IPE) within programs in higher education institutions can foster collaboration among students from different health professions (47). Authors have previously defined IPE as the practice of students from different health professions learning ‘from, with and about each other’ in preparation for health care work (48–51). Learning together actively could be executed for example through integrating interprofessional problem-based learning (PBL) into the curricula, in order to improve interprofessional cohesion and patient care (52). This has been shown to drive co-constructive discussions between students from various backgrounds (53).

In 2019, the State of Qatar inaugurated its first Dental College at Qatar University, employing PBL as the primary teaching method (54). This innovative approach aligns with the principle of IPE, advocating for the inclusion of students from various health disciplines, including Dental, PH and NU in collaborative learning endeavors. The aforementioned principle of IPE suggests the inclusion of dental, PH and NU students in learning activities together and the potential benefits this could bring to students in terms of exchanging knowledge and experiences, nevertheless, the effectiveness should be investigated further. While the potential benefits of such integration are apparent in terms of knowledge and experience exchange, the effectiveness of this approach warrants further investigation. The identified gaps in knowledge, particularly regarding the meaning of dental plaque and the number of teeth in a human dentition, highlight areas where targeted educational interventions are needed. To address these gaps, it is recommended that the curricula for Public Health and Nutrition programs incorporate specific modules on oral health, emphasizing practical knowledge and the importance of oral hygiene. Interactive workshops, hands-on demonstrations, and the inclusion of OH education in planned educational sessions can enhance understanding and retention of OH information. Additionally, leveraging social media and digital platforms to disseminate OH information could engage students effectively, given their high usage of these technologies. These interventions can equip future healthcare professionals with the necessary knowledge to promote OH in their professional practice.

Based on the findings of this study, future research could explore several avenues to enhance our understanding of OH knowledge, attitudes, and practices among university students. One potential study could involve a longitudinal design to assess changes in OH knowledge, attitudes and practices (KAP) over time, particularly after the implementation of targeted educational interventions. Another area for future research could involve comparative studies including both male and female students to identify gender-specific differences and inform more tailored educational strategies, as the current institutional policy provided a practical constraint on the participant pool available for this study. While this focus allows for a deep understanding of OH knowledge, attitudes, and practices among female students, it inherently limits the generalizability of our findings to the broader student population, including males. Additionally, exploring the impact of integrating OH education into various health-related curricula on students’ professional practice and patient outcomes would provide valuable insights into the long-term benefits of such educational initiatives.

## Limitations

This was a cross-sectional study and therefore did not lend itself to hypothesis testing. The sample size though adequate, was not as balanced between the two groups as would have been hoped for.

Content validity of the questionnaire was undertaken. However, factorial analysis would have enabled a more thorough look at questionnaire responses. The intake of students at the colleges were restricted to female students, hence comparative analysis was not possible, and findings cannot be generalized. The absence of male participants means that potential gender differences in OH knowledge and behaviors are not captured in this study. Moreover, no comparison was made in the students’ responses over the 4 years of training in PH and NU, as it could be assumed that students in the last years of training have more knowledge than those in the first years of training. Future research with larger sample sizes could include both male and female participants to provide a more comprehensive understanding of oral health KAP across genders. Moreover, analyzing the frequency of tooth brushing could provide evidence-based support of influence on OH.

## Conclusion

In conclusion, this study sheds light on the OH knowledge, attitudes, and practices among female public health and nutrition students. While participants demonstrated generally good knowledge regarding oral health, there were notable gaps, particularly in understanding dental plaque. Despite recognizing the importance of OH in overall well-being, certain practices fell below expectations, such as the frequency of dental visits. The findings highlight the necessity for increased awareness and the potential integration of oral health-related courses into the curriculum of public health and nutrition programs. Tailored interventions are crucial to address the specific needs and challenges encountered by different student populations. By acknowledging these variations in OH perceptions and experiences, healthcare professionals and educators can develop targeted strategies to promote awareness, improve hygiene practices, and enhance overall well-being among students. Furthermore, future research should delve into the underlying factors contributing to these differences and evaluate the effectiveness of interventions designed to mitigate them. This holistic approach can contribute to the development of comprehensive OH initiatives tailored to the needs of diverse student populations.

## Data Availability

The raw data supporting the conclusions of this article will be made available by the authors, without undue reservation.
